# Subclinical Myocardial Dysfunction Demonstrated by Speckle Tracking Echocardiography in Children with Euthyroid Hashimoto’s Thyroiditis

**DOI:** 10.4274/jcrpe.galenos.2019.2018.0273

**Published:** 2019-11-22

**Authors:** Emine Azak, Seyit Ahmet Uçaktürk, İbrahim İlker Çetin, Hazım Alper Gürsu, Eda Mengen, Utku Pamuk

**Affiliations:** 1 University of Health Sciences, Ankara Child Health and Diseases Hematology Oncology Training and Research Hospital, Clinic of Pediatric Cardiology, Ankara, Turkey; 2 University of Health Sciences, Ankara Child Health and Diseases Hematology Oncology Training and Research Hospital, Clinic of Pediatric Endocrinology, Ankara, Turkey

**Keywords:** Hashimoto’s thyroiditis, myocardial function, speckle tracking echocardiography, children

## Abstract

**Objective:**

Thyroid hormones have an important role in the regulation of the cardiovascular system. The aim of this study was to investigate the presence of subclinical myocardial dysfunction in children with euthyroid Hashimoto’s thyroiditis (eHT) without evident heart disease using tissue doppler imaging (TDI) and speckle tracking echocardiography (STE) methods.

**Methods:**

TDI and STE were peformed in 50 children with eHT and in 35 healthy children. To assess myocardial velocities and time intervals, including peak systolic velocity (S_m_), peak early diastolic velocity (E_m_), peak late diastolic velocity (A_m_), isovolumetric contraction time (IVCT), isovolumetric relaxation time (IVRT) and ejection time (ET), TDI was performed at the base of the interventricular septum (IVS) and in the left and right ventricles (LV and RV, respectively). Analysis of myocardial deformation by STE including strain (S) and strain rate (SR) was performed globally in two planes, longitudinal (L) and mid-circumferential (C) in LV [LV global longitudinal strain (LVGLS), LV global longitudinal strain rate (LVGLSR), LV global circumferential strain (LVGCS), LV global circumferential strain rate (LVGCSR)] and RV [(RV global longitudinal strain (RVGLS), RV global longitudinal strain rate (RVGLSR)].

**Results:**

Among TDI parameters, ET at LV and IVS were significantly lower, IVRT and myocardial performance index at LV and IVS were significantly higher in the eHT group compared to controls (p=0.001). There were no significant differences in S_m_, E_m_, A_m_ and IVCT values between patients and controls. LVGLS, LVGLSR, LVGCS and LVGCSR values were significantly lower in patients than controls (p=0.01). There was a negative correlation between thyroid antibody levels and LV global longitudinal and circumferential strain and strain rate values (TPO-Ab and Tg-Ab between LVGLS, LVGLSR, LVGCS and LVGCSR; r=-411, p<0.001; r=-541, p<0.001; r=-430, p<.0.001; r=-502, r<0.01 and r=-397, p<0.001; r=-473, p<0.001; r=-519, p<0.001; r=-421, p<0.00, respectively).

**Conclusion:**

The results show that myocardial function in children with eHT is impaired in the absence of any clinical symptoms and that conventional echocardiography is inadequate to determine these changes.

**What is already known on this topic?**

The cardiovascular system is affected by abnormal thyroid hormone levels, which are detected in overt hyperthyroidism, hypothyroidism and states of subclinical thyroid dysfunction. However, the effect on cardiovascular function in thyroid patients who are euthyroid on treatement is unclear.

**What this study adds?**

Impairment of global left ventricle myocardial function is present in children with Hashimoto’s thyroiditis who are euthyroid on treatment and conventional echocardiography is inadequate to determine these changes. In this study, we demonstrated that speckle tracking echocardiography is a useful method in the early detection of myocardial dysfunction in children with euthyroid hashimoto’s thyroiditis.

## Introduction

Abnormal thyroid hormone levels in states of overt hyperthyroidism, hypothyroidism and subclinical thyroid dysfunction affect many biological functions including the cardiovascular system. However it is unclear if, changes in cardiac performance associated with overt thyroid dysfunction are the result of alterations in myocardial contractility or loading conditions or both remains unclear (1,2,3,4). Hashimoto’s thyroiditis is the most commonly encountered, acquired thyroid function disorder in children (5). However, the cardiovascular effects of euthyroid Hashimoto’s thyroiditis (eHT) are unclear. Current studies indicate that eHT may be associated with left and right ventricular myocardial dysfunction. It has been suggested that the cardiovascular effects of eHT might be related to the abnormal inflammatory state associated with autoimmunity as well as to endocrine effects (3,4,6,7,8,9,10).

The aim of this study was to evaluate myocardial function using tissue doppler imaging (TDI) and speckle tracking echocardiography (STE) methods in children with eHT with no obvious heart disease. STE is a method that has been recently developed that evaluates parameters of myocardial deformation, even in the absence of clinical signs of abnormal cardiac function (3,6,8). To our knowledge, there is no study which used both TDI and STE to assess both left ventricle (LV) and right ventricle (RV) function in children with eHT.

Assessment of myocardial parameters in eHT with normal LV ejection fraction (EF) may be informative because these echocardiographic indices assess the multidirectional function of the entire myocardium of the LV and RV.

## Methods

In this study, TDI and STE for both LV and RV were performed in children with eHT and in healthy children. The relationship between changes in left ventricular myocardial mechanics and laboratory markers was also investigated.

### Study Population

This cross-sectional and case-controlled study was conducted from January to December 2016. A total of 50 patients with eHT, aged 5-18 years were recruited from the Pediatric Endocrinology Outpatient Clinics of Ankara Children’s Hematology and Oncology Research and Training Hospital. Detection of goiter was the reason for referral to the endocrinology department. The diagnosis of Hashimoto’s thyroiditis was based on estimation of thyroid stimulating hormone (TSH), free triiodothyronine (fT3), free thyroxine (fT4), antithyroglobulin antibody (Tg-Ab) levels and antithyroid peroxidase antibody (TPO-Ab) levels, supported by ultrasonographic findings of thyroid parenchymal heterogeneity. The study included patients who presented to the pediatric endocrinology outpatient clinic with goiter and were diagnosed as eHT and remained euthyroid by clinical and laboratory findings for at least six months of follow-up. Inclusion criteria were positive antibodies against thyroid TPO-Ab and/or Tg-Ab, euthyroid function (TSH <6.0 mU/L, normal values for fT3 and fT4), Hashimoto’s thyroiditis duration ≥6 months, normal LV EF (≥60%), good metabolic control. Patients with a normal TSH levels and positive thyroid autoantibodies were evaluated once more after six months, and were included in the study if their TSH levels were still within normal levels and two thyroid autoantibodies were positive. None of the patients had any other systemic or autoimmune disease and were not on any medication. Patients who had congenital and organic heart disease, arrhythmia and anemia were excluded from the study. There were no children receiving thyroid hormone replacement therapy because hormone levels were within normal ranges in all patients.

The control group consisted of 35 age and gender matched, healthy children who had presented to the pediatric cardiology clinic for evaluation of innocent heart murmurs. The same physical examinations and laboratory investigations were performed in the control group. Children with abnormal findings on laboratory testing, electrocardiograms and echocardiography were excluded.

### Clinical Data

Anthropometry and blood pressure measurements were carried out in both eHT patients and controls. Goiter staging was performed according to the definition proposed by Perez et al (11). After 12 hours of fasting, venous blood samples were taken to measure fT3, fT4, TSH concentrations and Tg-Ab and TPO-Ab levels by Elecsys Analyzer (Roche, Mannheim, Germany) using the electrochemiluminescence immunoassay method. Reference ranges used for thyroid hormones were: fT3: 0.18-0.44 ng/dL; fT4: 0.8-2.2 ng/dL; TSH: 0.27-4.2 µ IU/mL; Tg-Ab: 0-4 IU/mL; and TPO-Ab: 0-9 IU/mL. Systolic and diastolic blood pressure were measured using a standard mercury sphygmomanometer after 20 minutes of rest.

### Thyroid Imaging Methods

Thyroid ultrasonography was performed by using a 10 MHz linear transducer (General Electric, Logic 7, Horten, Norway) by experienced radiologists. In all patients, findings of thyroid ultrasonography (size of thyroid glands, parenchymal echogenicity) were recorded. Thyroid volume was calculated for each lobe by using the following formula: height x width x depth x 0.529 (11). Thyroid gland volume and volume standard deviation score were calculated using ÇEDD Çözüm Software (TPEDS Metrics) (12,13). Thyroid gland volume was assessed by comparison with age- and sex-adjusted thyroid volumes established by the World Health Organization (14).

### Echocardiographic Examination

### Conventional Echocardiography

A commercially available ultrasound system (iE33, Philips, The Netherlands, Eindhoven), equipped with a broadband (1-5 MHz) S5 transducer was used to obtain 2D grayscale harmonic images at a frame rate of 60-80 frames per second (frames/s). Two-dimensional and M-mode echocardiography was used to measure left ventricular end-diastolic and end-systolic diameter, end-diastolic septal and posterior wall thickness, EF and shortening fraction (FS), according to the guidelines of the American Society of Echocardiography (15).

### Tissue Doppler Imaging

TDI measurements were performed on the basal septum and on the LV and RV lateral walls. Filters were set to exclude high frequency signals. Gain was minimized to obtain clear signals, and images were recorded at a velocity of 100 mm/s. The maximal systolic myocardial velocity (S_m_), and early and late diastolic myocardial velocity (E_m_ and A_m_) were measured. The isovolumetric contraction time (IVCT) was calculated from the beginning of QRS in the echocardiogram until the beginning of the S_m_ wave. Isovolumetric relaxation time (IVRT) was calculated from the end of the S_m_ wave until the beginning of the E_m_ wave. Ejection time (ET) was measured from the beginning to the end of the S_m_ wave. Mean values were recorded by averaging the results of three consecutive measurements. The myocardial performance index (MPI; Tei index), which is a doppler-derived index including both systolic and diastolic time intervals to generate a combined index of global ventricular function, was calculated according to the formula; (IVCT+IVRT)/ET (16).

### Speckle Tracking Echocardiography

All two-dimensional STE analyses were performed by the same investigator to avoid inter-observer variability. Myocardial deformation parameters (S and SR) were measured using commercially available software (QLAB Advanced Quantification Software, version 6.0, TMQ, Philips Medical systems, Best, The Netherlands, Eindhoven) on standard 2D grayscale LV images from the standard apical 4-chamber view (AP4) for longitudinal strain and standard parasternal short axis at the papillary muscle level (PML) for circumferential strain. Two consecutive beats synchronized to a continous electrocardiography (ECG) were recorded with frame rate set to >60 frames/s. The data were transferred to the QLAB software system for off-line analysis. The endocardial borders were identified manually to include the entire myocardium in all view areas. The following peak systolic LV and RV STE parameters were measured:

- LVGLS: Left ventricular global longitudinal strain at AP4,

- LVGLSR: Left ventricular global longitudinal strain rate at AP4,

- LVGCS: Left ventricular global circumferential strain at PML,

- LVGCSR: Left ventricular global circumferential strain rate at PML,

- RVGLS: Right ventricular global longitudinal strain at AP4,

- RVGLSR: Right ventricular global longitudinal strain rate at AP4.

### Statistical Analysis

SPSS for Windows (version 18; SPSS Inc., Chicago, IL, USA) was used for the statistical analysis. Kolmogorov-Smirnov test was used to analyze the distribution of continuous variables. Numeric variables are expressed as the mean±standard deviation. Chi-square analysis were used to compare continuous and categorical variables between groups. Comparisons of demographic data and echocardiographic parameters between patients and controls were performed using Mann-Whitney U test for non-normally distributed variables. A difference was considered statistically significant at a p value of <0.05. Spearman’s correlation coefficient was used to disclose possible correlations between thyroid volumes, Tg-Ab, TPO-Ab and all echocardiographic data.

The number of patients that should be included in the study was calculated by Russ Lenth’s power analysis software (www.stat.uiowa.edu/~rlenth/Power/). The control group of the study was smaller than the study group. For this reason the Power analysis was based on the “mean LVGLS levels” as main outcome, when the mean levels for the study and control groups was given as -23 and -25, respectively, and a common standard deviation of 3. The difference between the two groups can be compared with 34 cases in each group (total 68 cases) using the Independent Samples t-test with an effect size of 0.7 (medium), a two-sided p value of 0.05, and a power of 81%.

## Results

### Clinical Characteristics of the Study Population

A total of 50 patients with eHT and 35 healthy controls were evaluated. The mean age of the patients was 12.5±3.2 years. Of the patients, 37 were girls (74%) and 13 boys (26%). There was no significant difference in age, gender and body mass index (body mass index; kg/m^2^) between the eHT group and the controls. Heart rate, systolic and diastolic blood pressure values were similar in both groups. No significant differences in fT3, fT4 and TSH levels were found between the groups. Compared to the control group, patients with eHT had significantly higher Tg and TPO antibody levels (p<0.001). A stage 1a goiter in 16 (32%) and stage 1b goiter in 34 patients (68%) were detected. The mean thyroid volume in the patient cohort (n=50) was 10.1±3.5 mL (range: 4.9-16.0 mL). The patient and control groups had normal ECG findings. Baseline characteristics and laboratory results of study groups are given in Table 1.

### Association of Thyroid Volume and Tg and TPO Antibody Levels

There was no correlation between Tg-Ab, TPO-Ab levels and thyroid volume in patients with eHT.

### Conventional Echocardiographic Findings

The eHT and control groups were not significantly different for LV end-diastolic diameter, diastolic thickness of the interventricular septum and LV posterior wall or for left ventricular FS and EF. FS and EF were within normal limits in both groups. Conventional echocardiographic findings are summarized in Table 1.

### Tissue Doppler Imaging Findings

TDI assessment of LV showed statistically significantly higher values of IVRT and MPI at IVS and LV in the eHT group compared to the control group. Additionally, ET values at LV were significantly lower in patients with eHT. There was no significant differences in S_m_, E_m_ and A_m_ values between the groups (p<0.05, Table 2). There were no significant differences in TDI values at RV in the eHT group compared to controls.

### Speckle Tracking Echocardiographic Findings

**Figure 1:**
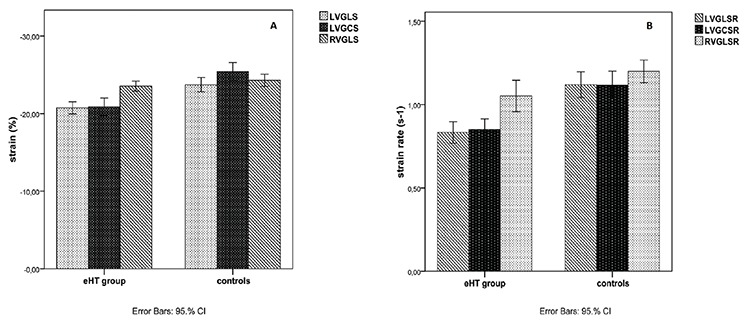
Myocardial strain and strain rate values. A) LVGLS: Left ventricle global longitudinal strain, LVGCS: Left ventricle global circumferential strain, RVGLS: Right ventricle global longitudinal strain. B) LVGLSR: Left ventricle global longitudinal strain rate, LVGCSR: Left ventricle global circumferential strain rate, RVGLSR: Right ventricle global longitudinal strain, RVGLSR: Right ventricle global longitudinal strain rate eHT: euthyroid Hashimoto’s thyroiditis

**Figure 2:**
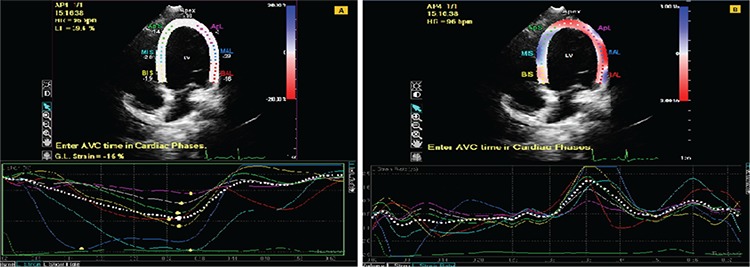
Two dimensional strain and strain rate analysis through speckle tracking echocardiography imaging of euthyroid Hashimoto’s thyroiditis. A) Strain analysis, B) strain rate analysis

The eHT group had statistically significantly lower LVGLS and LVGCS values compared to controls. Also LVGLSR and LVGCSR values were significantly lower in the eHT patients. There were no statistically significant differences for RVGLS and RVGLSR values between patients and controls (Figures 1, 2, Table 3).

### Association of LV STE Parameters with Laboratory Markers and Thyroid Volume

**Figure 3:**
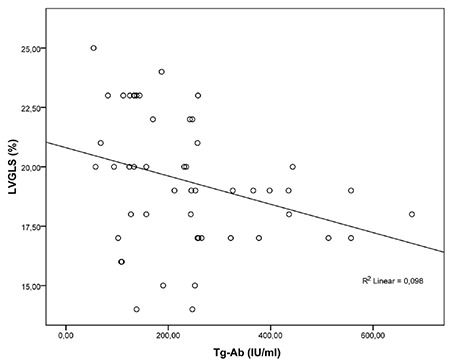
The correlation between myocardial strain and Tg-Ab levels LVGLS: left ventricle global longitudinal strain, Tg-Ab: antithyroglobulin antibody

**Figure 4:**
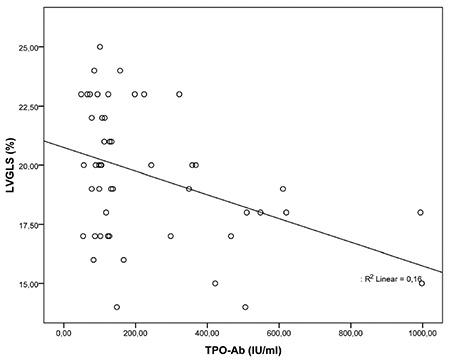
The correlation between myocardial strain and TPO-Ab levels LVGLS: left ventricle global longitudinal strain, TPO-Ab: antithyroid peroxidase antibody

There was a negative correlation between Tg-Ab, TPO-Ab levels and LV global longitudinal and circumferential strain and strain rates (Figures 3, 4, Table 4). However, there was no correlation between Tg-Ab, TPO-Ab levels and RVGLS and RVGLSR. In addition, thyroid volume showed no significant correlation with left ventricular global longitudinal and circumferential strain and strain rates (Table 4).

## Discussion

Thyroid hormones exert significant effects on the cardiovascular system. Thyroid dysfunction is a condition which affects cardiac performance and it is related with the risk of heart failure. There are two main thyroid hormone receptor genes in the human heart. The receptors are encoded by two genes (TRα and TRβ), each of which undergoes alternate splicing to generate receptor subtypes with differing tissue distributions. The TRα has been shown to play an important role in regulation of cardiac genes. T3 is the biologically active form of thyroid hormone and effects the heart by increasing some of these genes (1,2,3,4,17,18,19,20). The impact of hyperthyroidism or hypothyroidisim on the cardiovascular system is well known. Hyperthyroid patients have an increased heart rate and stroke volume that result in a high cardiac output state. An increased prevalence of LV hypertrophy and increased LV contractility has been reported in patients with overt hyperthyroidism. In contrast hypothyroid patients have low heart rate and low stroke volume that results in low cardiac output. Additionally, overt hypothyroidism has been reported as associated with decreased cardiac contractility (15,16,17,18). A recent study showed that long-term thyroid hormone replacement in euthyroid patients after myocardial infarction significantly improved LV contractility (21,22,23).

The cardiovascular effect of eHT in adults have been extensively studied (6,10). However, reasons for changes in cardiac performance in euthyroid patients remain unclear and children with eHT may be at higher risk for developing cardiovascular diseases (10,24).

Deleterious effects of eHT on the LV and RV systolic and diastolic functions have been reported, indicating that Hashimoto’s thyroiditis affects myocardial function regardless of thyroid hormone levels (6,10). Conventional echocardiography and TDI can be used to evaluate both the systolic and diastolic function of the heart in hypothyroid and hyperthyroid state, but the diagnostic value of conventional echocardiography is limited in the early phase of cardiac dysfunction (25,26). The impact of Hashimoto’s thyroiditis on myocardial systolic and diastolic functions has been studied using TDI in some previous studies (5,6,10). In some recently reported studies, evaluation of left ventricular systolic function with conventional echocardiographic method were found to be normal but left ventricular systolic dysfunction was demonstrated by TDI and STE methods even in euthyroid stage of patients with Hashimato’s thyroiditis. Furthermore STE is found to be a more sensitive parameter that shows left ventricular function (6,16,20). In this study, TDI of the IVS showed significant longer IVRT and shorter ET, consequently a higher Tei index. Additionally, LV-Tei index was significantly increased in the eHT group and this increase is related more to prolongation of IVRT than to shortening of ET, thus reflecting the impairment in both systolic and diastolic functions. Tei index was found to be more sensitive in the evaluation of diastolic relaxation than parameters such as deceleration time and E/A ratio, as previously reported (23,24). Akgul et al (6) also reported an impairment of global LV performance in adult patients with eHT. They showed an impaired Tei index and TDI-derived diastolic parameters despite normal findings by conventional echocardiography.

Recently new imaging techniques have been introduced to evaluate myocardial mechanics. STE is a novel echocardiographic method and strain and strain rate obtained by STE provides an opportunity for quantitative assessment of cardiac function. STE can be used as a diagnostic method in the early stages of many cardiomyopathic diseases. Myocardial global longitudinal strain values ​​were shown to have reduced without any changes in conventional echocardiographic parameters (27). Subclinical myocardial dysfunction can be detected early by TDI and STE methods. STE is a more recent technique that provides a global approach to ventricular myocardial mechanics and cardiac deformation and appears to be a sensitive diagnostic method for early detection of myocardial involvement in asymptomatic patients (6,8,27). We are not aware of any studies that have investigated myocardial functions by STE in children with eHT. This present study aimed to detect myocardial involvement in the euthyroid stage of HT. Recent studies showed that eHT is associated with an increased pulsed-wave velocity, independent of arterial atheromatosis indicating a direct impact of this disorder on arterial stiffening (6,7,16,28,29). Akgul et al (6), concluded that heart rate variability is significantly reduced in Hashimoto’s thyroiditis patients as a result of cardiac autonomic dysfunction, even at the euthyroid stage. Therefore, mechanisms that may explain cardiac autonomic and functional changes in eHT are probably related with abnormal cytokine profiles. However, the molecular, physiological and clinical evidence is still controversial (2,17,18,28,29).

The underlying pathophysiologic mechanism leading to the cardiovascular effects of eHT have not yet been fully understood. Some mechanisms leading to cardiovascular system involvement have been reported previously in patients with Hashimoto thyroiditis.

Firstly, the majority of eHT patients are in a state of slow, progressive thyroid dysfunction. It is widely acknowledged that most of these patients will progress to a state of hypothyroidism. Thus, it may be hypothesized that the insidious progression to thyroid dysfunction in Hashimoto’s thyroiditis may be responsible for the cardiovascular adverse effects, even in subjects with normal serum thyroid hormone levels (7,28,29).

Secondly, the spectrum of clinical signs may change during the course of HT. Thus, eHT patients may have been hypothyroid or hyperthyroid previously even though they are euthyroid at the time of assessment and LV and RV functional changes might be due to a previous hypothyroid or a hyperthyroid phase (24). In the present study, we have shown that Hashimoto’s thyroiditis is associated with subclinical LV systolic and diastolic dysfunction, even when the patients are euthyroid. Conventional echocardiography does not exclude subclinical left ventricular wall motion abnormalities in patients with eHT. The myocardial dysfunction could be identified as a reduction of LV global and circumferential strain and strain rate and TDI derived Tei index. Accordingly, we showed that children with eHT had a significantly lower left ventricular strain and strain rate values, as well as decreased IVS and LV Tei index values compared to controls.

Thirdly, the autoimmune state associated with Hashimoto’s thyroiditis could be the responsible for cardiovascular changes, rather than the effects of secreted hormones. Autoimmunity induced endothelial dysfunction and inflammation may have an important role in the pathogenesis of cardiovascular conditions seen in these patients, such as hypertension, atherosclerosis and myocardial dysfunction (6,10,28,29). In Hashimoto’s thyroiditis, it has been reported that goiter may be present either due to lymphocytic infiltration of the thyroid gland or to increased TSH levels caused by hypothyroidism. However, there is a controversy regarding the role of antibodies in the development of goiter (30). In our study, no significant correlation was detected between serum level of thyroid antibodies and thyroid volume. In addition, no significant correlation was detected between thyroid volume and STE parameters. In these patients, there is typical heterogeneous hypoechogenic parenchyma on thyroid sonography. In our cases, serum TSH concentrations were within normal range. In fact, it may be more relevant to evaluate the relationship between serum antibody level and heterogeneity of thyroid gland parenchyma rather than volume. Moreover, we found a correlation between serum thyroid antibody levels and STE paramaters in our patients. Subclinical systolic and diastolic dysfunction of the LV appeared to be significantly related to TPO-Ab and Tg-Ab levels. So, our findings suggest that the autoimmune state associated with Hashimoto’s thyroiditis, rather than abnormal secreted hormone concentrations, could be responsible for the cardiovascular effects.

### Study Limitations

Our study has several limitations. Firstly, the relatively small number of patients could be considered as a limitation. Secondly, we did not investigate the effect of thyroid replacement therapy on LV and RV functions in eHT patients.

Further clinical research is needed with larger patient groups to investigate the mechanisms on myocardial dysfunction with normal LV EF in eHT patients.

## Conclusion

In this study, it was demonstrated that STE is useful in the early detection of myocardial dysfunction in patients with eHT. Impairment of global LV myocardial function is present in children with Hashimoto’s thyroiditis who are euthyroid and replacement therapy naive. In addition, conventional echocardiography was inadequate to detect these changes. It is important to increase the data available in this field, particularly prospective data. There is also a need for additional prospective data related to cardiac function in eHT patients. Subclinical myocardial dysfunction in the early disease may be considered as an indication for initiation of thyroid replacement treatment even in euthyroid patients.

## Figures and Tables

**Table 1:**
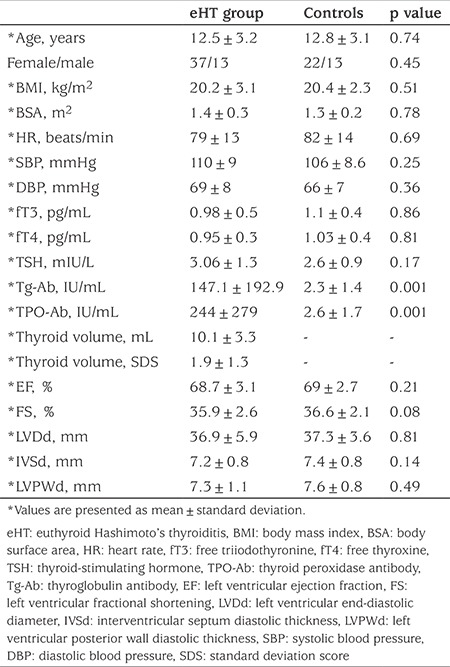
Demographic and laboratory variables, thyroid volume and conventional echocardiographic findings in the study groups

**Table 2:**
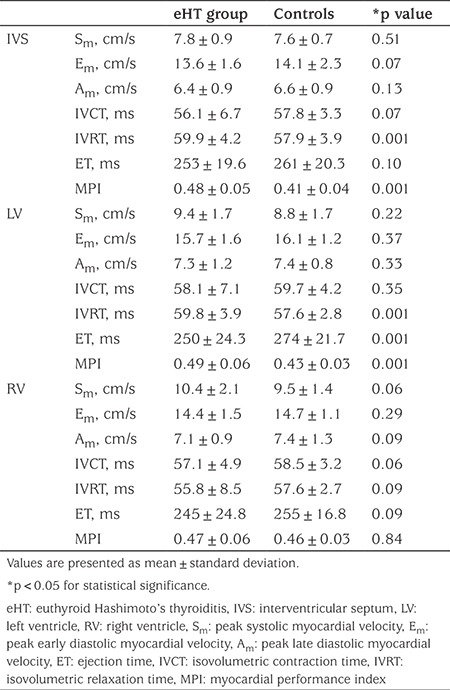
Tissue doppler echocardiography measurements in the study groups

**Table 3:**
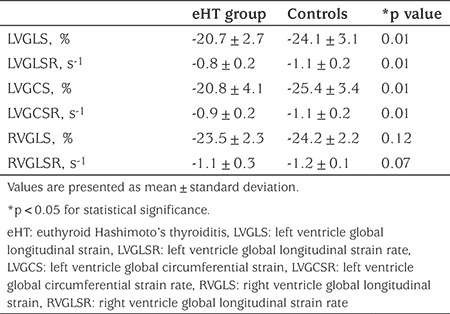
Speckle tracking echocardiography measurements in the study groups

**Table 4:**
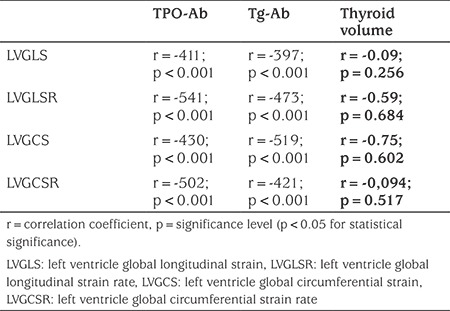
Correlation between left ventricular speckle tracking echocardiography parameters and thyroid antibody levels and volume in euthyroid Hashimoto’s thyroiditis group
